# Phosphorylation of caspase-9 at Thr125 directs paclitaxel resistance in ovarian cancer

**DOI:** 10.18632/oncotarget.23133

**Published:** 2017-12-08

**Authors:** Mi Ran Byun, Jin Woo Choi

**Affiliations:** ^1^ Department of Pharmacology, College of Pharmacy, Kyung Hee University, Seoul 02447, Republic of Korea

**Keywords:** ovarian cancer, paclitaxel-induced resistance, caspase-9, CDK1

## Abstract

Although paclitaxel is routinely prescribed for the treatment of epithelial ovarian cancer (EOC), paclitaxel resistance is common in EOC and correlates with short survival of patients. A previous pharmacogenomic study revealed the importance of cyclin-dependent kinase 1 (CDK1) activity in a response on paclitaxel. However, a subsequent research showed that the expression level of CDK1 failed to show significant correlation with delayed apoptosis and patient survival. Rather, the expression and phosphorylation of capase-9, the downstream target molecule of CDK1, appeared to determine drug resistance. Our results suggest that treatment with the CDK1 inhibitor alsterpaullone reduces phosphorylation of caspase-9. Its phosphorylation level was dependent on CDK1 activity and it directs paclitaxel resistance. This observation was reproducible in xenografted tumors. Thus, the regulation of caspase-9 may be a novel therapeutic strategy to reverse paclitaxel-induced resistance in ovarian cancer cells.

## INTRODUCTION

Epithelial ovarian cancer (EOC) is one of the most lethal gynecologic diseases in the world [1]. Although at the initial stage of the disease, the first line chemotherapy like paclitaxel is responsive, the cancer cells acquire drug resistance at their late stage, which leads poor prognosis of EOC patients [2]. Identification of these resistance mechanisms may offer valuable insight to design strategies that modify the chemoresponse of cancer cells and reverse drug resistance. Myriads of regimens have been reported as therapeutic combinations to regulate drug resistance of cancer cells, and several strategies of genetic modulation have been suggested with the potential to switch the resistance [2–4].

Among them, paclitaxel resistance can be explained partially by β-tubulin-dependent mechanism, which is plausible as paclitaxel directly target microtubule and cell cycle [5]. Nevertheless, paclitaxel resistance remains unresolved. It seems to need more details of mechanism until a clinical use. CDKs forms the heterodimeric complex with cyclin proteins and the active conformation directs many transcription factors and leading to regulation of cell division. By the reasons, functional disorder of CDK causes neurodegeneration with abnormal cell death. More frequently, uncontrolled CDK activity is found in cancer cells out of microenvironmental control. A protective role of CDK to suppress unexpected cell death has been reported. CDK1 directly phosphorylates caspase-9 and the phosphorylated caspase-9 at Thr125 turns itself inactive. This link between CDK1 and caspase-9 is thought to suppress unnecessary cell death during eukaryotic cell [6, 7]. It is likely because among twenty CDKs, CDK1 is only essential CDK for cell cycle regulation in mammals, whereas CDK2 and 3, which exhibit high homology with CDK1, are dispensable in control of cell cycle [8]. However, rather high expression of CDK1 expression can cause paclitaxel resistance in cancer cells. A previous study showed that alsterpaullone, a CDK1 inhibitor, reversed drug resistance *in vitro* and *in vivo* [9]. Here, we intend to define the details of CDK1-induced drug resistance and trace the molecules that act downstream of CDK1. Although CDK1 was reported as a druggable target to reverse paclitaxel-induced chemoresistance, we newly suggest that caspase-9 is a rate-limiting step to control the acquirement of paclitaxel resistance.

## MATERIALS AND METHODS

### Cell lines and materials

Ovarian cancer cell lines A2780, HeyA8, SKOV3, A2780-CP, HeyA8-MDR and SKOV3-TR were a kind gift from Dr. Jeong Won Lee (Samsung Medical Center, South Korea). A2780 was established from ovarian endometroid adenocarcinoma tumor and A2780-CR is cisplatin-resistant cell line derived from the parental A2780. HeyA8-MDR is multi drug resistant ovarian cancer cell line induced by paclitaxel. SKOV3, the parental cell of SKOV3-TR was originally derived from ovarian serous cystadenocarcinoma and SKOV3-TR was a taxen-resistant type induced by paclitaxel treatment [10]. All ovarian cancer cells were incubated in RPMI-1640 containing 10% FBS (Gibco) and 250 ng/ml gentamycin. To maintain chemoresistance, SKOV3-TR and HeyA8-MDR cells were supplemented with 150 and 300 ng/mL paclitaxel. Paclitaxel, alsterpaullone, propidium iodide (PI), and Hoechst 33342 were purchased from Sigma Aldrich. Cisplatin was obtained from LC Laboratories (MA, USA).

### Cell viability assay

Cell viability was measured by using an MTT assay (Promega, Ltd.) according to the manufacturer’s method. Cells were seeded at 5 × 10^3^ cells in 96-well culture plates and treated with cisplatin for 12 h. The cells were then incubated with 5 mg/mL MTT for 4 h, removed growth medium, 150 µL of solubilization solution were added and incubated for 4 h. The absorbance of each well was measured at 570 nm wavelength. For the cell death analysis, cells were stained with 10 µM Hoechst 33342 and 1 µM PI for 20 min. Cells were then washed twice with serum-free media and stained cells were detected with a fluorescence microscope (Nikon, Japan).

### Western blot analysis

Cells were lysed in radioimmunoprecipitation assay buffer containing 20 mM Tris-HCl [pH 7.5], 150 mM sodium chloride, 1 mM disodium ethylenediaminetetraacetic acid, 1 mM ethylene glycol tetraacetic acid, 1% Triton, 2.5 mM sodium pyrophosphate, 1 mM β-glycerophosphate, 1 mM sodium orthovanadate and protease inhibitor (Cell Signaling Technology, Inc.). Total cell lysates were denaturated in 5x SDS sample buffer, separated on SDS-PAGE gels and transferred to PVDF membranes. The membranes were incubated with antibodies against caspase-9 (BD Biosciences), phospho-caspase-9/Thr125 (Abcam), CDK1 and β-actin (Santa Cruz Biotechnology).

### Animal experiments

All animal experiments were performed in compliance with the institutional guidelines approved by the Institutional Animal Care and Use Committee at Wonkwang University (WK 2014-06-BR-006). Xenograft model was prepared by injection of 2 × 10^6^ HeyA8-MDR cells into the flank of 8-week-old C57BL/6 mice. 3 weeks later, the mice were injected with 5 mg/kg alsterpaullone or 0.9% saline before administration of paclitaxel and administered with 7.5 mg/kg paclitaxel during another 3 weeks.

### Immunohistochemistry

To analyze xenografted tumors, tumor tissues were fixed, dehydrated and embedded in paraffin. The paraffin embedded tissues were then sectioned at a thickness of 5 μm and blocked endogenous peroxidase activity with 1% hydrogen peroxide for 5 min. The tissue sections were incubated with antibodies against caspase-9 and caspase-9/Thr125 and developed with DAB (3,3′-diaminobenzidine) for 7 min. Nuclei were counterstained with Mayer’s hematoxylin, slides were mounted and imaged under a microscope (Nikon Eclipse 80).

## RESULTS

### Relevance of CDK1 in paclitaxel sensitivity

**Figure 1:**
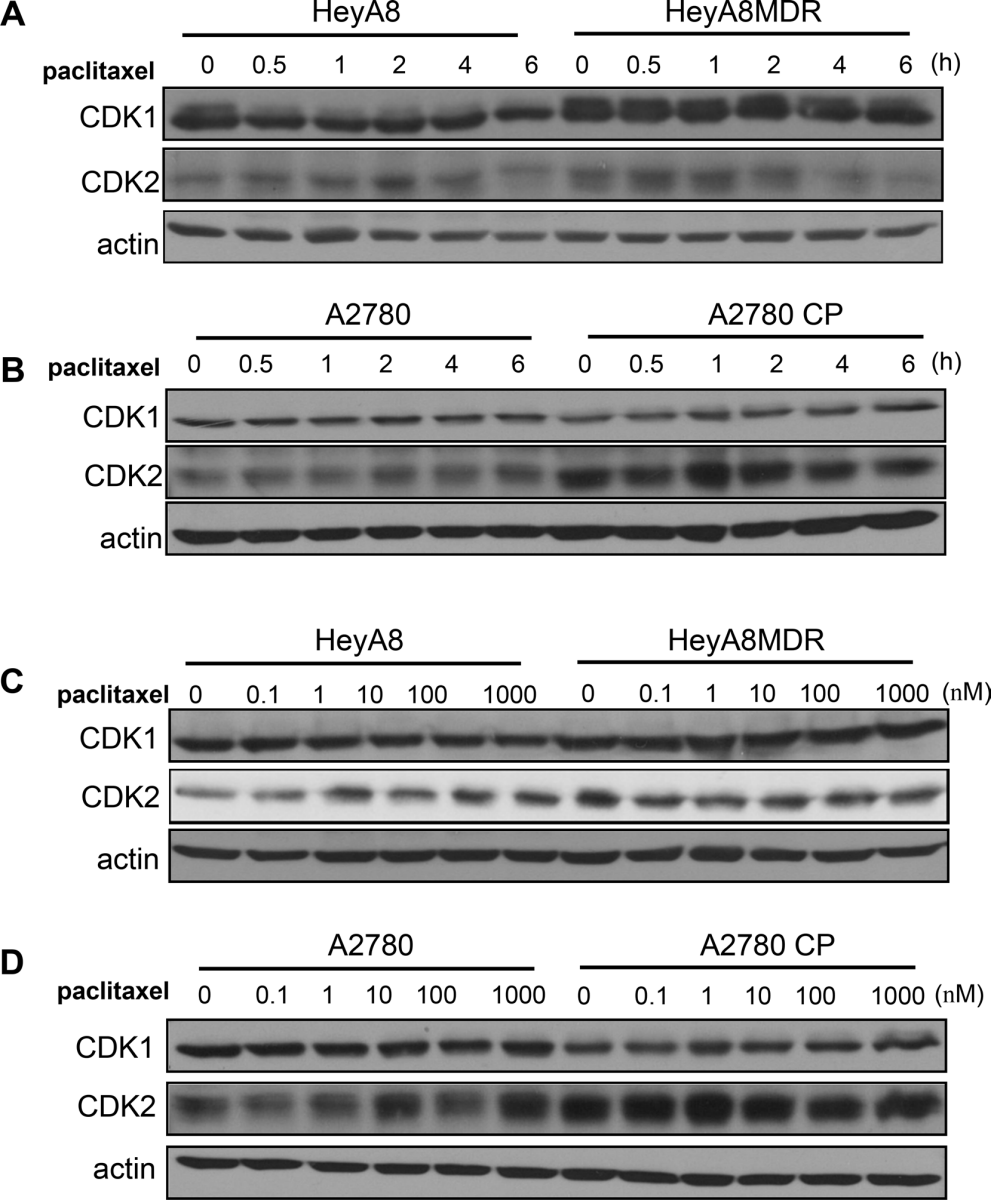
Expression of CDK1/2 in paclitaxel-resistant cells (**A**–**B**) CDK1 and CDK2 levels were analyzed by western blot. Actin was used as a loading control. (**C**–**D**) CDK1/2 levels were detected by western blot analysis after treatment of cells with different concentration of paclitaxel.

We compared chemosensitive and chemoresistant cells for CDK1 expression levels using western blot analysis. CDK1 expression was commonly upregulated in the paclitaxel-resistant HeyA8-MDR cells as compared with drug-sensitive cells (Figure 1A). As this phenomenon relies on the response of cells to drug treatment, we monitored cells in a time-dependent manner after paclitaxel treatment. Although a fluctuation in CDK1 expression was observed based on drug treatment time, CDK1 basal level was significantly higher in paclitaxel-resistant cells. However, no difference was observed in CDK1 level between cisplatin-sensitive A2780 and cisplatin-resistant A2780-CP cells (Figure 1B). In A2780 cell pairs, CDK2 expression, rather than CDK1, was significantly increased in A2780-CP cells. This result of the CDK1 expression was observed in a dose-dependent manner (Figure 1C and 1D) and the CDK1 and CDK2 protein level was reported to be almost similar during the time course. Taken together, CDK1 appears to be specifically upregulated in paclitaxel-resistant cells.

### Paclitaxel resistance is mediated by the phosphorylation of caspase-9

**Figure 2:**
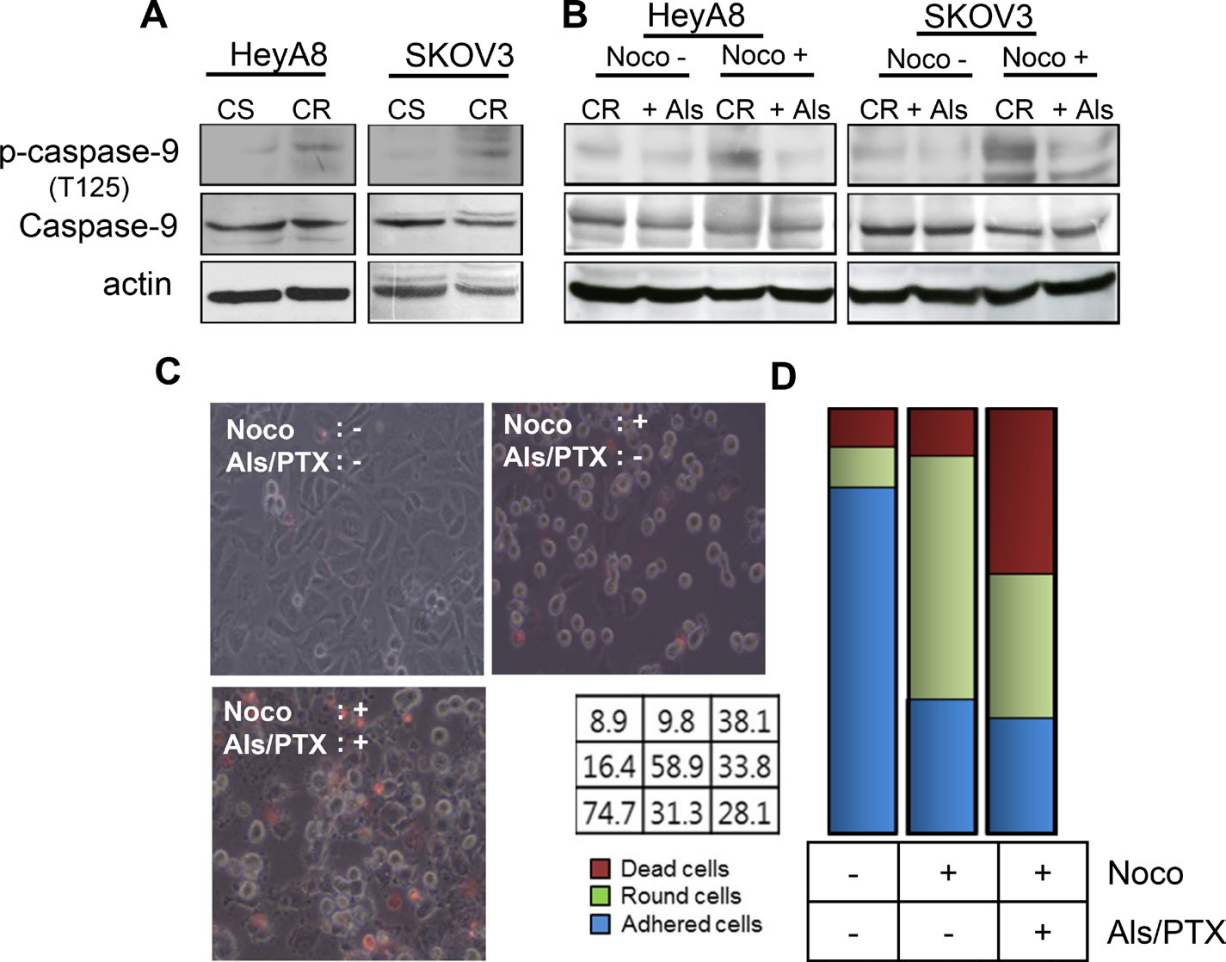
CDK1 mediates paclitaxel-induced chemoresistance through caspase-9 (**A**) Total caspase-9 and phospho-caspase-9 levels were analyzed by western blot. Actin was used as a loading control. CS, chemosenstive; CR, chemoresistant. (**B**) HeyA8-MDR and SKOV3-TR cells were treated with nocodazole to arrest cells in their mitosis. Noco, nocodazole; Als, alsterpaullone; PTX, paclitaxel. (**C**) Cell death was compared between three experimental groups: alsterpaullone-pretreated cells (upper left), alsterpaullonepretreated cells with added nocodazole (upper right), and alsterpaullone-pretreated cells with added paclitaxel and nocodazole (lower left). Administration of paclitaxel was followed by nocodazole treatment. (**D**) Populations of adherent (blue) and floating (green) cells are shown. Dead cells were stained with red dye.

The phosphorylation of caspase-9 via CDK1 kinase activity mediates the suppression of cell death during mitosis [11]. Therefore, we investigated whether the reversal of paclitaxel resistance by alsterpaullone treatment occurs through the modulation of capase-9 phosphorylation. Basal levels of phosphorylated caspase-9 were higher in paclitaxel-resistant cells, HeyA8-MDR

and SKOV3-TR, as compared to their chemosensitive counterparts (Figure 2A). Repeated western blot analysis showed a low intensity band, which may be attributed to the phosphorylation of caspase-9 during the metaphase of cell cycle. Thus, we first attempted to synchronize the cell cycle in G2/M phase by nocodazole treatment. Analysis of cells during metaphase revealed a successful increase in the phosphorylation levels of caspase-9 (Figure 2B, third lane of each panel). In the absence of nocodazole, phospho-caspase-9 levels failed to show any significant change even after alsterpaullone treatment. Thus, CDK1 inhibitor alsterpaullone inhibited the phosphorylation of caspase-9 in chemoresistant HeyA8-MDR and SKOV3-TR cells during nocodazole-pretreated metaphase (Figure 2B). We tried to validate whether the reversal of paclitaxel resistance in the alsterpaullone-pretreated cells is caused by cell population in metaphase. During metaphase, most cells display a rounded morphology. Based on these morphological changes, we confirmed the cells arrested in metaphase. After treatment with nocodazole, the population of floating cells increased to 58.9%. Upon treatment with paclitaxel, the population of dead cells increased from 9.8% to 38.1%, while the proportion of floating cells sharply decreased from 58.9% to 33.8%. The proportion of adhesive cells was unchanged (Figure 2C and 2D). This data support our hypothesis that alsterpaullone suppresses the activity of CDK1 in metaphase cells.

### Correlation between caspase-9 phosphorylation and paclitaxel-induced cell death

Although CDK1 may induce phosphorylation of caspase-9, no correlation was observed between CDK1 expression level and survival of patients with ovarian cancer. We tested if the expression of CDK1 may shorten patient survival, given its close association with drug resistance. Gene expression data were selected from 567 patients who had a survival record from The Cancer Genome Atlas (TCGA). Based on CDK1 expression level, the data were divided into 85 patients in top 15% and another 85 patients in bottom 15%. In contrast to our expectation, no significant difference was observed between the two groups (Supplementary Figure 1).

**Figure 3:**
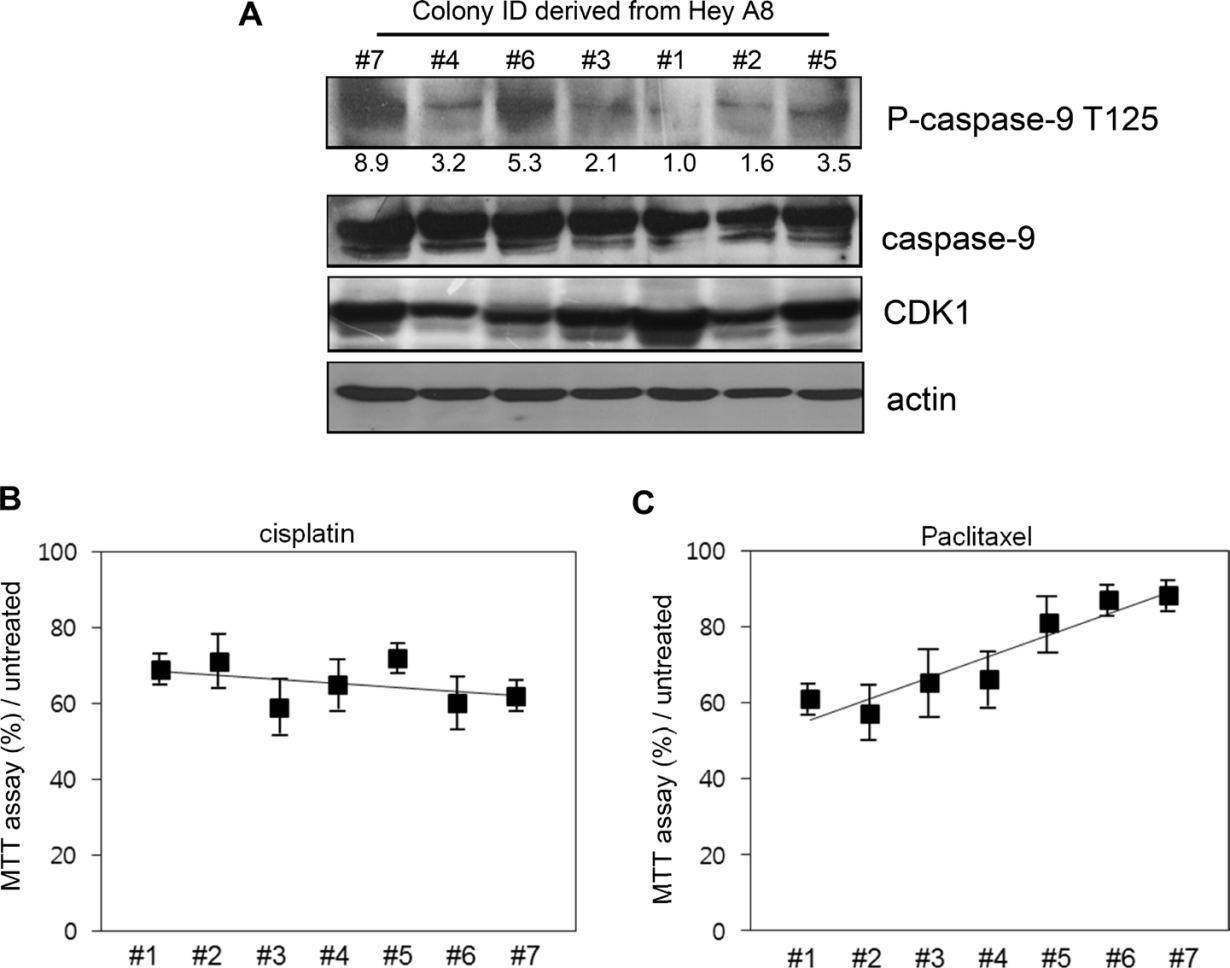
Correlation between caspase-9 phosphorylation level and resistance to cell death (**A**) The colonies with different phosphorylation level were selected after low-dose paclitaxel treatment and cultured for 7 additional days. The ID number was conferred based on the phosphorylation level detected with western blot analysis. Relative density of phospho-caspase-9 was analyzed by Image J 3.0. (**B**) Different cells were treated with cisplatin for 12 h and cell viability assessed using MTT assay. (**C**) Cells were treated with paclitaxel and cell death assessed using MTT assay.

To determine if the phosphorylation level of caspase-9 correlates with the resistance to cell death, we treated drug-sensitive HeyA8 cells with 10 µM (low dose) paclitaxel. In these cells, caspase-9 phosphorylation was induced by paclitaxel at different degrees. We numbered the degree of phosphorylation from 1 to 7 according to the measured density (Figure 3A), 1 being smaller than 7 at phosphorylation.

We compared the cell propensity in the presence of 200 µM (high dose) paclitaxel. The same number of cells was spread on the plates and the cell death was analyzed. Although cell death was similar between the cells upon cisplatin treatment (Figure 3B), MTT assay percent value showed a negative correlation with the phosphorylation level of caspase-9 (Figure 3C).

### Caspase-9 is critical for paclitaxel-induced cell death

**Figure 4:**
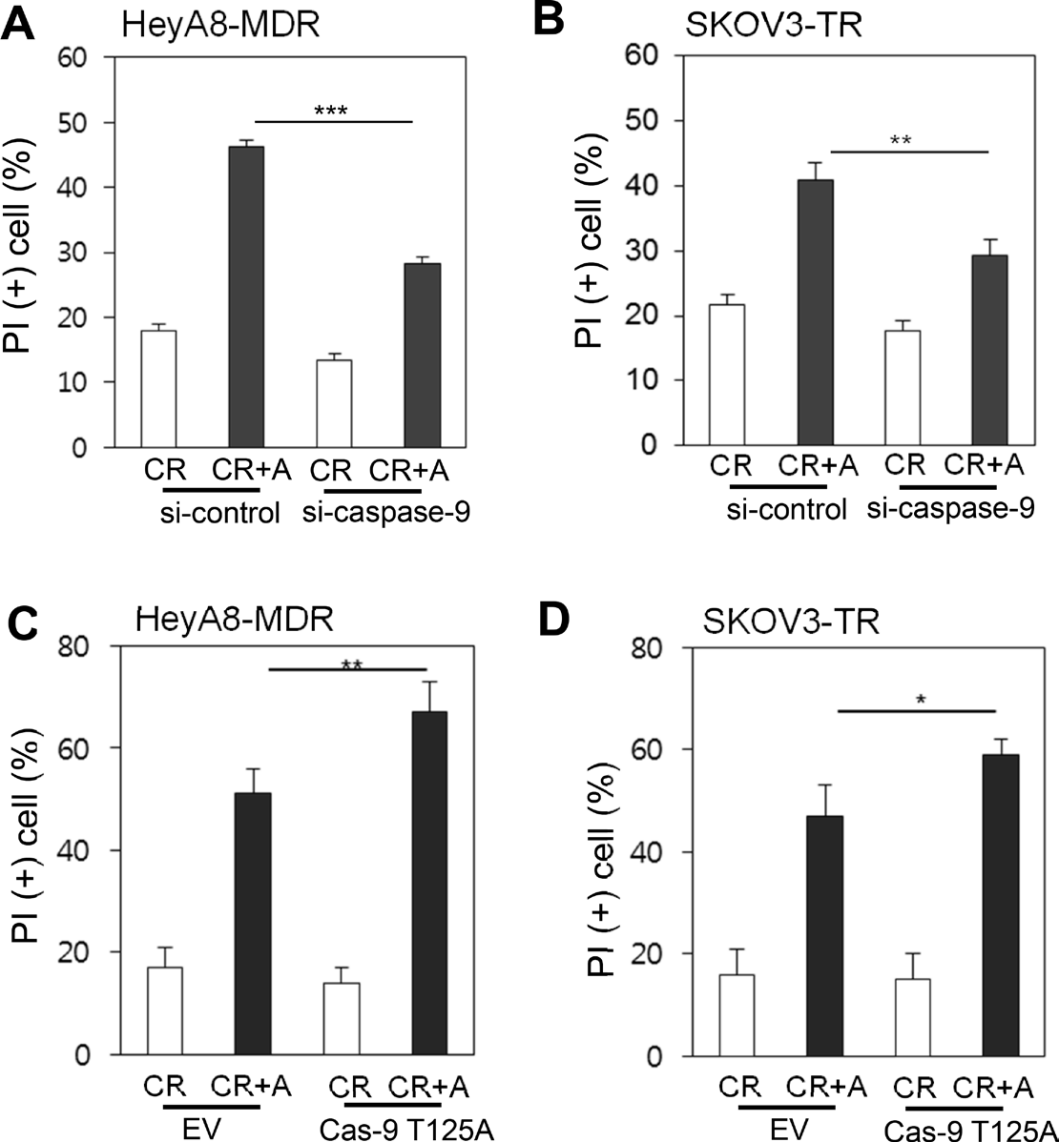
Caspase-9-dependent cell death (**A**–**B**) siRNA against caspase-9 was administrated in chemoresistant paired cell lines, HeyA8 and SKOV3. A mixture of random siRNAs was used as a control. (**C**–**D**) Cells were transfected with T125Amutant caspase-9, followed by treatment with alsterpaullone and paclitaxel. ^*^, *p* < 0.05;^**^, *p* < 0.01;^***^, *p* < 0.001.

To directly evaluate whether caspase-9 is critical for alsterpaullone-mediated reversal of paclitaxel chemoresistance, we targeted caspase-9 with siRNA in HeyA8-MDR and SKOV3-TR cells. Suppression of caspase-9 with siRNA compensated for alsterpaullone-mediated chemoresistance reversal by 20% and 14% in HeyA8-MDR and SKOV3-TR cells, respectively (Figure 4A and 4B). We used T125A site mutant vector of caspase-9, which shows defective phosphorylation. T125A-transfected cells showed significantly increased cell death upon alsterpaullone/paclitaxel treatment in HeyA8 and SKOV3 cells (Figure 4C and 4D).

### *In vivo* validation of alsterpaullone-mediated chemoresistance reversal

**Figure 5:**
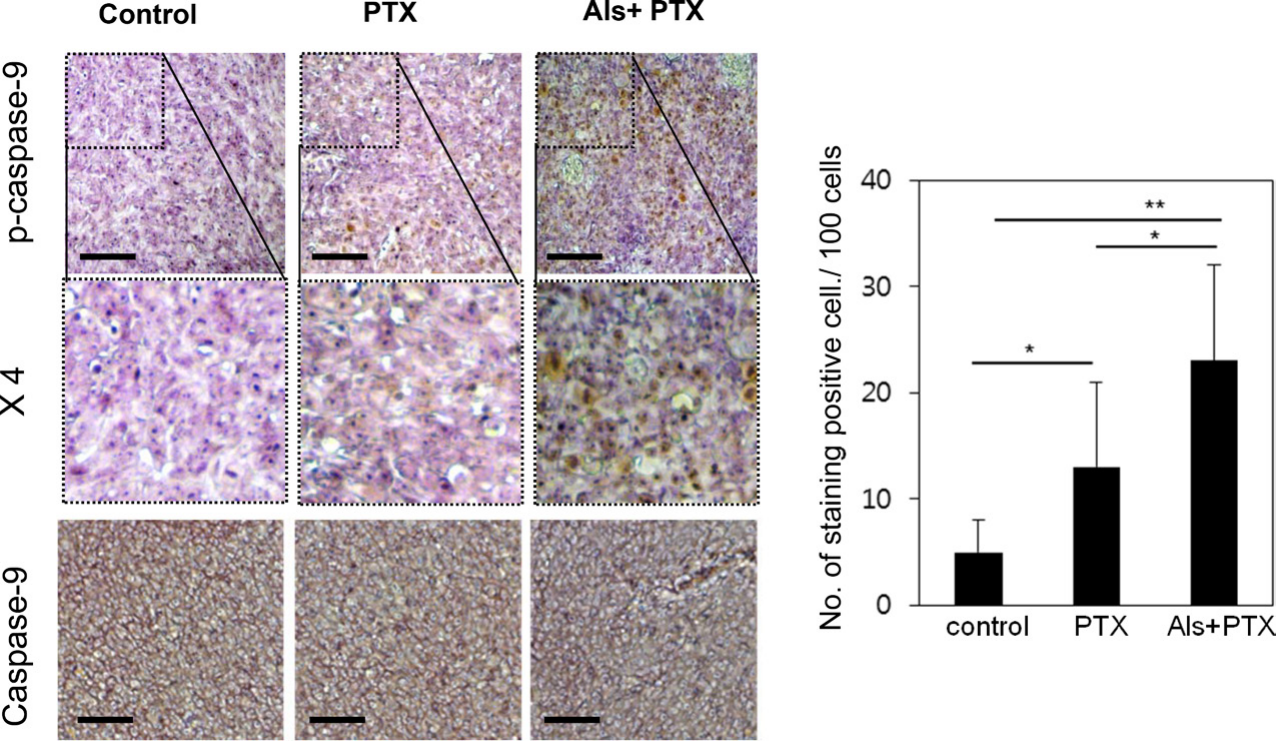
*In vivo* validation of alsterpaullone-mediated chemoresistance reversal Immunohistochemical analysis of molecular markers in control, paclitaxel-treated, and alsterpaullone-pretreated tumors. Representative images of tumors in each treatment group. PTX, paclitaxel; AP, alsterpaullone.^*^,*p* < 0.05;^**^, *p* < 0.01.

We injected HeyA8-MDR cells in the flank region of mice. Three weeks before administration of paclitaxel, either 0.9% saline or alsterpaullone (5 mg/kg) was intraperitoneally injected every other day. At week 7, the tumors were isolated and subjected to immunohistochemical analysis. Phospho-caspase-9 level was lower in tumors treated with alsterpaullone as compared with the untreated tumors (Figure 5).

### Inhibition of caspase-9 phosphorylation by alsterpaullone

**Figure 6:**
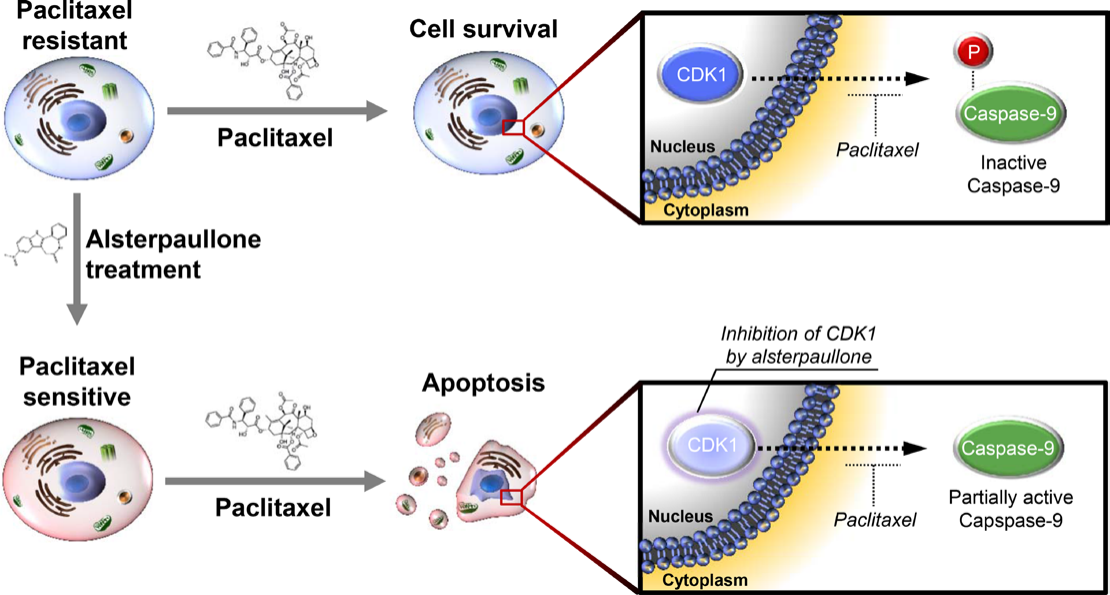
Schematic diagram explaining the mode of action of alsterpaullone Phosphorylation of caspase-9 indicates its inactive form. In the lower panel, the light color of CDK1 is intended to express the relatively reduced activity of CDK1.

The activity of CDK1 is reported to increase aberrantly as cancer cells acquire resistance to paclitaxel. During this process, caspase-9 is readily phosphorylated, thereby blocking paclitaxel-mediated cell death (Figure 6, upper panel). Continuous alsterpaullone treatment for more than 2 weeks reduced CDK1 activity. As a consequence, cells again showed a partial response to paclitaxel (Figure 6, lower panel).

## DISCUSSION

Cancer may be curable, if cancer cells fail to acquire drug resistance. Ovarian cancer is one of the most referred cancer types to demonstrate drug resistance. Of the various resistance-causing drugs, we studied the effect of paclitaxel as a combinatory or single chemical drug for clinical treatment of ovarian cancer [12, 13]. Paclitaxel inhibits the disassembly of microtubules during mitosis and is a naturally attractive target for anticancer therapy. In ovarian cancer, paclitaxel resistance is easily observed in clinics and is the cause of poorer survival. In our previous study, we suggested that CDK1 inhibitor alsterpaullone may reverse paclitaxel resistance. During mitosis, CDK1 phosphorylates and cleaves caspase-9, leading to its inactivation and activation of caspase 3 and 7 [14]. Thus, the overexpression of caspase-9 even in drug-sensitive cancer cells deems cells vulnerable to aberrant apoptosis under normal condition. Otherwise, suppression of caspase-9 inhibits apoptosis (Figure 4A and 4B). Thus, caspase-9 must be a critical factor for the initiation of cell death. Cancer cells with acquired resistance may show different phases of cell death. In paclitaxel-resistant cells, caspase-9 seems inefficient to induce caspase cascade for cell death. As shown in Figure 3A, the total caspase-9 level was not proportional to phosphorylated caspase-9 level and cellular viability. In addition, patients with high expression of caspase-9 showed a short survival (*p* = 0.1; Supplementary Figure 1B). Although CDK1 expression level failed to show precise correlation with drug resistance, the level of phospho-caspase-9 is a better predictor of paclitaxel resistance. Thus, phosphorylation of caspase-9 is a good marker of paclitaxel resistance.

Phosphorylation of caspase-9 at Thr125 may be also mediated by extracellular signal-regulated kinase 1 (ERK1) [15]. These results suggest that the phosphorylation of Thr125 on caspase-9 may be an important mechanism through which growth factors and survival signals activate the ERK–mitogen-activated protein kinase (MAPK) pathway. This phenomenon may explain the constitutive overexpression of Erk1 observed in tumorigenesis. Thus, phosphorylation of caspase-9 at Thr125 may be the target of various oncogenic kinases [16] and a valuable marker for the prediction of cancer patient prognosis. Although alsterpaullone was selected as an effective adjuvant therapy for paclitaxel-treated patients, other phosphatases for caspase-9 may be more effective for their anti-cancer or drug resistance reversal effect.

## SUPPLEMENTARY MATERIALS FIGURE


