# Supplementary material for: Phosphorylation of caspase-9 at Thr125 directs paclitaxel resistance in ovarian cancer
Source: Oncotarget. 2017 Dec 8;9(1):1041–7. doi: 10.18632/oncotarget.23133 (PMC5787417; doi:10.18632/oncotarget.23133)
Supplement: Supplementary file 1 [file oncotarget-09-1041-s001.pdf]

Phosphorylation of caspase-9 at Thr125 directs paclitaxel resistance in ovarian cancer

SUPPLEMENTARY MATERIALS

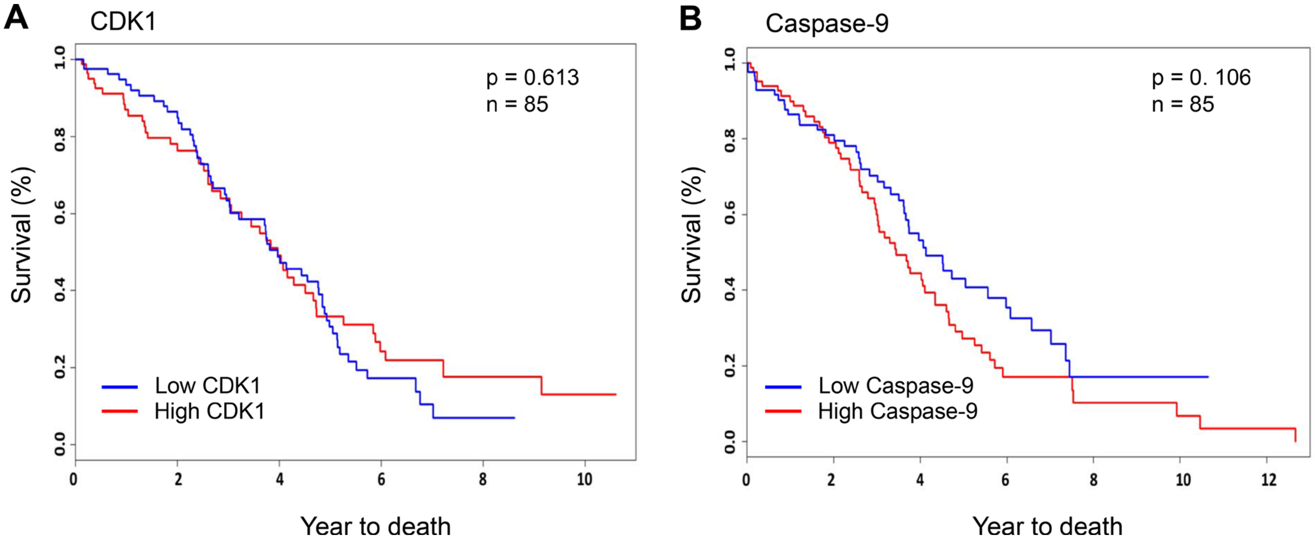

Supplementary Figure 1: Correlation analysis between CDK1 or caspase-9 expression level and patients survivals. (**A**) We tested if the expression of CDK1 may shorten patient survival, given its close association with drug resistance. Gene expression data were selected from 567 patients with a survival record from The Cancer Genome Atlas (TCGA). Based on CDK1 expression level, the data were divided into 85 patients in top 15% and another 85 patients in bottom 15%. In contrast to our expectation, no significant difference was observed between the two groups. (**B**) The patients with > 15% caspase-9 expression showed shorter survival after diagnosis.
